# Classification of unintentional injury prevention practices for infants and young children at home: developmental process and associations with other variables in Japanese families

**DOI:** 10.1186/s40359-025-02770-5

**Published:** 2025-04-25

**Authors:** Yasuo Kojima

**Affiliations:** Chukyo University, Nagoya, Japan

**Keywords:** Unintentional injuries, Infants and young children, Accident prevention practices, Developmental processes, Risk avoidance skills

## Abstract

**Background:**

Infants and young children often experience unintentional injuries at home, which are a significant public health concern worldwide. This study investigated the contents and types of accident prevention practices for unintentional injuries in families of infants and young children using a Japanese sample.

**Methods:**

A total of 875 mothers with children between the ages of 6 months and 6 years and 11 months participated in the online survey. Respondents answered items regarding specific strategies to prevent unintentional injuries at home, temperament, independence of life habits and their children's behaviors, and parenting attitudes. Measures to prevent unintentional injuries were examined using multiple correspondence analysis, and cluster analysis was performed on the sample scores extracted from the correspondence analysis. Data on children's temperament, life habits and behaviors, and mothers' child-rearing practices were analyzed using factor analysis and scored. Finally, multinomial logistic regression analysis was conducted with the cluster classification of strategies to prevent unintentional injuries as the objective variable and other variables as explanatory variables.

**Results:**

From the correspondence and subsequent cluster analyses, three clusters were extracted: the first cluster (*n* = 517) was the *preventative group* that kept objects that could lead to injuries away from children in advance; the second (*n* = 173) was the *constantly restrictive group* that explicitly prevented children from approaching or contacting dangerous places or objects; and the third cluster (*n* = 185) was the *case-by-case restrictive group* which was attentive to each effort to discourage children's contact with dangerous places. Cluster 1 was more common in the 0-year-old age group, Cluster 2 in the 1- and 2-year age groups, and Cluster 3 in the 4–6-year age groups. A multinomial logistic regression analysis revealed that the more independent the children were regarding life habits and behaviors, the more likely they were to be classified in the second cluster, and the more parents had an encouraging attitude toward their children to take on challenges, the more likely they were to be classified in the third cluster.

**Conclusion:**

The results of this study confirmed the general process by which countermeasures against children's unintentional injuries at home proceed from (1) the phase in which parents protect their children by keeping potentially hazardous objects away from them. (2) The phase in which parents prevent unintentional injuries by blocking contact and accessing hazardous objects and places. (3) The phase in which parents open some hazardous environments to their children but simultaneously monitor their children's behavior and respond to prevent injuries on a case-by-case basis. The data also showed that child-related variables (self-reliance on children's life habits and behavior) and parent-related variables (mothers' child-rearing attitudes) were associated with the countermeasures taken by the parents.

**Supplementary Information:**

The online version contains supplementary material available at 10.1186/s40359-025-02770-5.

## Background

Unintentional injuries refer to traffic injuries, drowning, poisonings, burns, and falls [1]. They are serious worldwide public health problems, directing mortality, morbidity, disability, and economic stress to the healthcare system and household level [2]. Statistics on unintentional injuries among children have been reported in many countries, and the World Health Organization (WHO) considers them to be a severe public health problem worldwide [1]. In Japan, unintentional injuries have long been one of the leading causes of death among children, ranking fourth at age 0, third at ages 1–4, and second at ages 5–9 [3]. The Ministry of Health, Labor, and Welfare [3] also reported that looking at the location of accidents involving preschool children, the younger the age, the higher the percentage of accidents occurring at home. Additionally, excluding traffic accidents, more than 90% of unintentional deaths at age 0 and 70% at ages 1–4 occurred at home.

Regarding non-fatal accidents, the percentage of children who experienced injuries that resulted in a hospital visit was 10%–20% for all preschoolers [4]; the percentage of children who experienced minor accidents that did not result in a hospital visit was 67.6% for 1.5-year-old children [5], and more than half of the preschoolers as a whole [6]. Regarding the location of accidents, including those that did not lead to a medical examination, 96.1% of accidents at age 0 and 76.7% of accidents at age 1 occurred indoors, mainly at home [7], with 68.8% of accidents at age 1.5-year-old occurring at home [8]. When children are old enough to walk, their exploratory skills, ability to move faster, and limited ability to identify and react to hazards [9, 10] often result in momentary lapses that can lead to major accidents. Therefore, adults play a major role in preventing and controlling their children's injuries [11].

Morrongiello [12] indicated that three factors are essential for children to avoid unintentional injury: (1) keeping the environment safe, (2) adult supervision, and (3) teaching children safety rules. It is believed that around the age of three, parents shift from the phase of environmental maintenance and monitoring to the phase of educating children about safety rules [13–15]. Especially in children aged < 3 years, accident prevention, countermeasures, and monitoring by parents are of the utmost importance [16–18]. Further, many accidents are said to be preventable through such strategies [19, 20]. Related to this, Rimsza et al. [21] noted that 90% of accidents in preschool children could be prevented for certain types of accidents. Additionally, Santagati et al. [22] reported that the children of their parents who were unaware that unintentional injuries could be avoided were more likely to suffer more injuries. Meanwhile, Ma et al. [23] noted that knowing that most unintentional injuries can be avoided motivates individuals to improve their home environment, leading to avoidance of unintentional injuries. Tanaka [24] noted that it is essential for parents to have knowledge of accidents and the development of infants and toddlers—to know what children become capable of as they age—and that it is essential to recognize precisely where and what hazards exist at home, and to take appropriate strategies to avoid these hazards.

With regard to taking countermeasures against unintentional injuries at home, parents are believed to be mainly involved in controlling and preventing accidents [25]. However, there are no fundamental data on the strategies used at home, when they are used, what methods are used, and especially when they cease to be used. Several studies have addressed the relationship between the presence or absence of strategies and their effects on the occurrence of unintentional injuries. However, little is known about how these measures change with children’s development, or how they relate to variables other than age.

In studies that have addressed children's unintentional injuries, several conceptual frameworks have been proposed for the factors involved in the occurrence of the injuries themselves (e.g., [10]). Moreover, models specific to unintentional injuries at home have also been suggested [26]. However, there is no adequate conceptual framework regarding the measures that parents should take to prevent unintentional injuries in children. Hence, this study focused on the two possible variables which are known to be related to the occurrence of unintentional injuries [26]: child-related and parent-related factors.

Based on Schwebel et al. [27] and Ma et al. [28], one potential factor related to unintentional injuries in children is their temperament. Available data showed that characteristics such as high activity, an inclination to prefer rough behavior, and a tendency to seek stimulation contribute to the occurrence of unintentional injuries [29–31]. Additionally, more inhibited children are less likely to be injured because they are more cautious about approaching novel or unusual objects [27, 32]. Furthermore, suppose the extent of children's self-reliance may be linked to unintentional injuries, measures of independence in life habits and behaviors were also exploratively incorporated into the variables. It was predicted that the more self-reliant the children were, the less protective the parents would be against unintentional injuries. Conversely, child-rearing attitudes of mothers were included as variables related to countermeasures against children's unintentional injuries. In Japan, the difference in time spent by parents on childcare remains substantial, with fathers spending one hour and five minutes, and mothers three hours and forty-five minutes per day [33]. Furthermore, existing data on the association between mothers'child-rearing attitudes and unintentional injuries have revealed that mothers who frequently monitor their children's behavior have fewer injuries [34, 35]. Based on Klommert [26], the mother's tolerance or the restrictiveness of the child's engaging in hazardous behavior was adopted as an indicator in this study.

Based on the above, this study aimed to (1) identify the types and content of injury prevention strategies employed by families with infants and young children and examine how they evolve, particularly the relaxation of monitoring and control measures as children grow older. (2) Explore the relationship between a child's intra-individual factors, such as temperament and independence in life habits and behaviors, and the mother's intra-individual factors, including parenting attitude, with the injury prevention strategies adopted. (3) Investigate the relationship between the individual factors of the mother and child, and the chosen prevention strategies for each age group of children.

## Method

### Study design and setting

A self-administered questionnaire was administered to parents of preschool children.

### Sample size

According to the latest statistical data available during this study [36], the number of children aged < 7 years in Japan was approximately 5,368,000. The required sample size was calculated with a confidence level of 95% and a margin of error of 5%, resulting in a sample size of 385 individuals using these data.

$$n=\frac{{Z}^{2}\cdot p\cdot \left(1-p\right)}{{E}^{2}}$$

$${n}_{\text{adj}}=\frac{n}{1+\frac{n-1}{N}}$$

*Z*: Z-score corresponding to confidence level (1.96 for 95% confidence interval).

*p*: the estimated proportion of the population (using the most conservative estimate of 50%, i.e., *p* = 0.5).

*E*: the margin of error (0.05 for ± 5%).

*N*: the population size (5, 368, 000).

In contrast, the necessary sample size was examined for the scale of injury prevention strategies, which is the primary topic of the study and contains the largest number of items. As described later, this scale consisted of 31 items, and the responses were treated as binary data in the analysis. Since it is generally considered that a minimum of five samples per option is necessary for the number of responses for each item, at least (2 × 5 =) 10 samples were considered essential for each item for this scale. However, this sample size is a minimum requirement, and, in practice, a larger sample size is preferable. Although there is no clear definition of how much sample is necessary for each item in the analysis of these types of scales, the reliability of the data is believed to be stable, with 20 samples per item in factor analysis [37]. Based on this criterion, 20 samples per item were collected (31 × 20 =) 620 samples. In an earlier survey by the author, which consisted of a similar number of items, approximately 30% of the data were not used in the analysis because they did not answer the questions faithfully. Therefore, expecting that 70% of the data could be used for analysis in this study, it was thought that at least (620/0.7 =) 885 respondents would be necessary. Finally, considering the possibility that more answers were unavailable as data, 1,000 respondents were invited to participate in the survey.

### Participants and procedures

The study was outsourced to Cross Marketing Co., Ltd., one of Japan's largest online research marketing companies with more than four million registered members. In December 2022, Cross Marketing Co., Ltd. provided the selected panels who met the following criteria: families with at least one child between the ages of 6 months and 6 years and 11 months who did not attend elementary school, with a URL that led them to the survey and invited them to respond. If there was more than one child within this age range, participants were asked to respond to the younger child. The company assigned the samples by dividing them by age (0, 1, 2, 3, 4, 5, and 6) and sex of the child (boys and girls), recruited them until 72 responses were collected for each cell (age × sex), and then stopped recruiting when 1,000 samples were collected. The number of respondents per age stage and number of sex-crossed cells were 71 and 72, respectively. Data were collected from December 23–26, 2022.

Mothers were selected as respondents. With regard to the control and prevention of unintentional injuries in children, mothers and fathers are considered equally responsible for practical countermeasures. However, the amount of time spent on parenting is unfortunately still distributed unevenly [38, 39] and is especially concentrated on mothers in Japan [33]. Some reports have indicated that mothers play a central role in this effort [40, 41]. Moreover, several previous studies on children's unintentional injuries asked mothers to respond to questionnaires [13, 26, 42, 43]; therefore, the mother was considered more appropriate as a respondent.

Families with twins (*n* = 13) were excluded from the analysis because it was unclear for which child was answered. Second, families of individuals living together who were non-relatives (*n *= 12) were excluded from the analysis. Additionally, those who responded to each question using one value on at least one of the scales used in this study were excluded from the analysis (*n* = 100) because they were considered not to answer the questions sincerely. The final analysis included data from 875 respondents.

### Measures

#### Demographic variables

Participants were asked about their family composition: age and sex of all children; age, educational background, and annual income of parents; regional characteristics in which they lived (rural, urban, or suburban); and type of residence (detached house, housing complex, or other).

#### Strategies to avoid unintentional injury

Based on 666 descriptions extracted from a previous study [44] conducted by the author and his colleagues on 275 parents with children under 1.5 years old, a 31-item scale was developed for the present study, which covered measures against unintentional injuries practiced in various locations at home. To confirm the validity of extending the target of these items to families with children < 7 years old, the consistency with the items described in the “Accident Prevention Handbook” published by the Children and Families Agency in Japan [45] was examined. Consequently, the validity of using this scale to examine injury prevention strategies in families with children aged < 7 years was confirmed (Supplemental file 1). The respondents were asked to choose one from the following three options:"1. currently practicing,""2. practiced in the past but not now,"or"3. never practiced in the past or present."In the analysis, the Answers of 2 and 3 were combined as"not implemented."

#### Temperament

Three items were selected from each of the four factors (negative emotional reactions, nervousness, adaptability, and extraversion) in the previous research's [46] temperament scale for infants and toddlers. This scale was developed based on the items of Toddler Temperament Scale (TTS) [47], Infant–toddler Social and Emotional assessment (ITSEA) [48], and Colorado Childhood Temperament Inventory (CCTI) [49] that have been widely used by researchers in many countries. The reliability and validity were confirmed to meet these criteria. The factor *negative emotional reactions* included items, such as “Short-tempered and angry,” *nervousness* included items, such as “Immediately notices when clothes get wet and wants to have clothes changed,” *adaptability* included items, such as “Be in a good mood when played with by strangers,” and *extraversion* included items, such as “Prefer to play with others than to play alone.” Responses were obtained on a four-point scale ranging from 1 being “not at all” to 4 being “very well.”

#### Independence of life habit and behavior

The measures developed by Yatagai and Takahashi [50] were used in this study. The original version of the questionnaire contained 46 items covering eight categories (eating, elimination, sleeping, hygiene and cleanliness, clothing, self-reliance, and social customs and routines). Duplicate items were excluded from this study. Furthermore, other items unrelated to life habits (e.g., playing with friends often, having hobbies to devote oneself to, and having a potential job in the future) were also excluded. Also, based on a previous finding [51], items with low implementation rates, even among school-aged children (e.g., cleaning one's room by oneself, managing one's allowance), were excluded. Finally, participants were asked to respond to 18 items: two related to eating, two related to sleeping, four related to hygiene and cleanliness, two related to clothing, two related to self-reliance, three related to social customs, and three related to social routines, using a three-point scale (1. not capable, 2. it depends on, 3. capable).

#### Mothers'parenting attitudes

Respondents were asked to respond to eight items from Sonoda [52], including four items related to respect for children's independent activities and four items on protective attitudes, on a four-point scale ranging from"1. not at all applicable"to"4. very applicable".

#### Unintentional injuries at home

Respondents were asked to reflect on the past year and whether their child had suffered a severe injury that required them to take them to hospital. Three options were given:"Yes,""No,"and"I do not remember."However, this item was excluded from the analysis.

### Statistical analyses

Data analysis was conducted using SPSS Windows software version 28.0. Regarding strategies to prevent unintentional injuries at home, multiple correspondence analysis was conducted based on the response (0: not implemented, 1: implemented), and then cluster analysis was conducted on the dimension scores extracted from the correspondence analysis. In the multiple correspondence analysis, the criterion for determining the number of dimensions was set to eigenvalues above 0.05, in line with a previous study [53]. Furthermore, low-frequency categories tend to be outliers owing to their distinct distributions from the average. Therefore, careful attention should be paid to the location of low-frequency categories in the scatterplots [54]. Hierarchical cluster analysis was performed using Ward’s method and squared Euclidean distance. The number of clusters was determined by the number of vertical lines in the dendrogram cut by a horizontal line that could transverse the maximum distance vertically without intersecting a cluster [55].

First, as a preliminary analysis, associations were examined among cluster classifications of strategies to prevent unintentional injuries and socioeconomic background factors (family structure, parental education, employment, and income), followed by the analysis of their association with children’s demographic variables, such as age range, birth order, and sex. Data on children's temperament and mothers'child-rearing practices were analyzed using confirmatory factor analysis, along with the findings of a previous study. Additionally, data on children's life habits and behaviors were not identified in the original study on background factors. Therefore, an exploratory factor analysis was conducted. Finally, a multinomial logistic regression analysis was conducted with the cluster classification of strategies to prevent unintentional injuries as the objective variable and other variables that had not been found to be associated in earlier analyses. There were no missing data, and data from all 875 participants were used for all analyses in this study. Statistical significance was set at *p* < 0.05.

### Ethical considerations

The study was conducted per the principles of the revised Declaration of Helsinki. The authors obtained approval from the Ethical Review Committee of Chukyo University before conducting the study. All the participants provided informed consent before commencing the online survey.

## Results

Table 1 shows the basic information of the participants. More than 90% of the participants were families consisting of parents and children, with most fathers living together in the same household. The sample size by child age was not highly biased, except for a slightly smaller sample of families with children aged 0 years (6–11 mo.). There was no sample size bias owing to sex or birth order. Most parents were university graduates (44.0% mothers, 45.0% fathers), followed by high school graduates (21.7% mothers and 20.8% fathers). While 32.0% of the mothers earned less than two million yen, 40.9% did not earn any income. In contrast, 33.7% of fathers earned between four and six million yen, 20.3% earned between six and eight million yen, and 16.2% earned between two and four million yen. While most mothers (47.0%) were housewives, 23.5% were employed part-time, and 25.1% were employed full-time. Most fathers (87.9%) were employed full-time.

**Table 1:** Demographic description of the sample

Variables	Category	N	%
**Family structures**			
Intact	Father living with family	795	90.9
	Father living away from family^1^	27	3.1
Single mother		53	6.1
**Children**			
Age	6―11 mo.	65	7.4
	12―23 mo.	109	12.5
	24―35 mo.	132	15.1
	36―47 mo.	143	16.3
	48―59 mo.	138	15.8
	60―71 mo.	145	16.6
	72 mo.―	143	16.3
Birth order	First-born	397	45.4
	Second or subsequent	478	54.6
Sex	Male	441	50.4
	Female	434	49.6
**Mother**			
Ecudational level	Junior high	20	2.3
	High	190	21.7
	Technical school	144	16.5
	Junior college	97	11.1
	University	385	44.0
	Graduate school	38	4.3
	Others	1	0.1
Annual income (Japanese yen)	None	358	40.9
	― 2,000,000	280	32.0
	― 4,000,000	108	12.3
	― 6,000,000	53	6.1
	― 8,000,000	15	1.7
	― 10,000,000	5	0.6
	― 15,000,000	1	0.1
	15,000,000 ―	0	0.0
	DK	55	6.3
Employment	Employed full-time	220	25.1
	Employed part-time	206	23.5
	Others	38	4.3
	None	411	47.0
**Father**			
Ecudational level	Junior high	16	1.8
	High	182	20.8
	Technical school	109	12.5
	Junior college	43	4.9
	University	394	45.0
	Graduate school	82	9.4
	Others	49	5.6
Annual income (Japanese yen)	None	4	0.5
	― 2,000,000	20	2.4
	― 4,000,000	134	16.2
	― 6,000,000	279	33.7
	― 8,000,000	168	20.3
	― 10,000,000	71	8.6
	― 15,000,000	21	2.5
	15,000,000 ―	4	0.5
	DK	126	15.2
Employment	Employed full-time	727	83.1
	Employed part-time	4	0.5
	Others	92	10.5
	None	4	0.5
	DK	48	5.5

^1^In Japan, fathers sometimes live apart from their families for work

### Measures to prevent unintentional injuries

Among the 31 measures to prevent unintentional injuries at home, item 26,"Install barriers on beds and keep children close to the wall to prevent them from falling in their sleep,"was excluded from the analysis because many families in Japan do not use beds but instead put futons on the floor and let children sleep on them. Additionally,"frequently wipe areas that children may touch to remove germs and debris"(item 30) were excluded from the analysis because the present study was conducted in 2022, which coincided with the COVID- 19 pandemic. It was considered more likely to have been undertaken as a measure against infection rather than to prevent unintentional injuries. The percentage of respondents who completed 29 items, excluding the above two, was calculated (Table 2).

**Table 2:** Percentage of participants who had implemented each of the measures to prevent unintentional injuries

Categories	Percentages
3. Keep an eye on the child as much as possible	80.11
22. Keep hot drinks and soup dishes away from the table's edge	76.00
19. Keep items that could cut or burn (scissors, knives, lighters, etc.) out of reach of the child	74.17
18. Keep items that could be put in the mouth (batteries, cigarettes, medicine, etc.) out of the child's reach	69.49
28. Put the lid on the toilet seat after using it	66.40
5. Lock the doors of places dangerous for the child to enter alone (bathrooms, toilets, balconies) when not in use	65.26
21. Remove any items (balcony chairs and stepladders) that could cause the child to fall if they climb on them	61.37
4. Lay a futon on the floor for the child to sleep on instead of a bed	60.11
6. Place a play mat on the floor	57.03
20. Move large items that could cause injury or burns (glass tables, rice cookers, humidifiers, etc.) to another location out of reach of the child	52.46
15. Drain the bath water immediately after use	52.00
24. Place chairs or other items in front of places that contain objects that could cause injury (kitchen sinks, drawers of shelves, etc.) so that the child cannot easily get to them	48.91
23. Keep cords of electrical appliances off the floor	47.77
2. Leave safe items only and allow the child to play in them	46.63
11. Attach a cover to the electric fan	45.26
9. Attach covers to electrical outlets	41.49
27. Place cushions, pillows, and blankets in places where they might fall or tumble from above	37.14
12. Install corner guards on tables and TV stands	36.80
10. Install child latches or other measures to prevent the child from opening shelves and drawers, among others, by themselves	35.09
8. Put things in front of places that could cause injury if they fell over (bookshelves, TVs, plants) and keep them away from the child	33.14
7. Install child safety guards	30.40
17. Remove tablecloths	29.83
16. Keep trash cans out of reach of the child	29.60
29. Turn off the main gas valve when not in use	26.17
25. Replace furniture that could cause injury	24.23
14. Install door stoppers	19.66
13. Put cushion stickers on fusuma, a door in Japanese rooms	17.83
31. Install a video monitor to see the child in another room	12.57
1. Allow the child to play in a baby circle	6.86

**Fig. 1:**
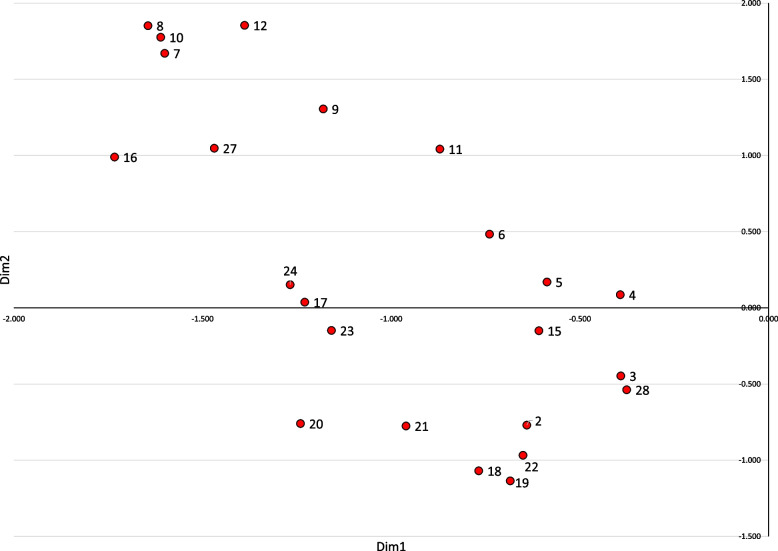
Results of multiple correspondence analysis (horizontal axis: 1 st dimension, vertical axis: 2nd dimension). (Figure legend) The numbers beside the data points correspond to the item numbers in Table 2

Next, multiple correspondence analysis was conducted using all the items shown in Table 2, based on the response data (0: not implemented, 1: implemented), with the number of dimensions fixed at two. The bottom five items (1, 31, 13, 14, and 25) were plotted in positions far from the overall distribution; therefore, these five items were excluded from the analysis. Analysis using these 24 items revealed eigenvalues of 0.286 and 0.062 for the first and second dimensions, respectively (cumulative proportion: 34.8%). Figure 1 shows that the larger the value for the first dimension, the more likely parents are to take precautions each time (e.g., 28 placing the lid on the toilet seat after use, and 15 draining the bathtub). The dimensions with smaller values indicated that the measures were performed only once and were maintained under the same conditions (e.g., 8 placing something in front of a dangerous object if it fell over and 7 installing child guards). Conversely, for the second dimension, those with large values concentrated on measures to explicitly block or close access to a particular room or place (e.g., 29 closing the main gas valve, 10 putting child locks on shelves and drawers, and 9 installing outlet covers). Contrastingly, those with small values concentrated on measures to keep away or remove the object—(e.g., putting away objects < 19 small objects, 20 large objects > that could cut or burn hands, 18 moving away objects that could be swallowed accidentally, and 21 removing objects that are dangerous to climb on).

**Fig. 2:**
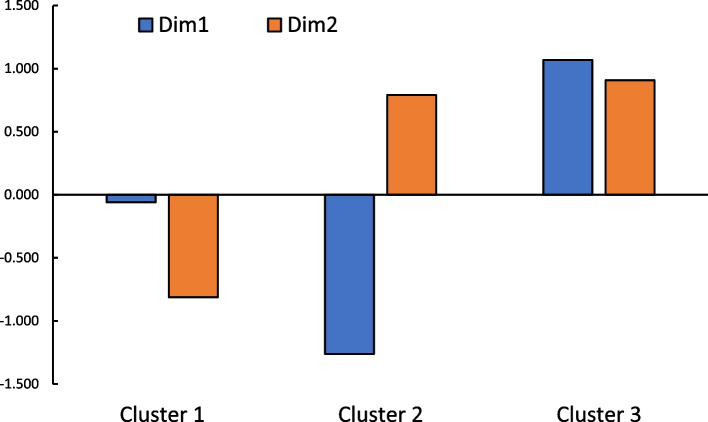
Characteristics of each cluster regarding measures to prevent unintentional injuries

Next, hierarchical cluster analysis using Ward’s method and squared Euclidean distance was performed on the sample scores of the first and second dimensions extracted by multiple correspondence analysis. The presence of three clusters was considered appropriate based on the shape of the dendrogram. These three clusters are characterized as follows (Fig. 2): Analysis using one-way analyses of variance was conducted with Clusters 1, 2, and 3 as independent variables and the sample scores of the first and second dimensions as dependent variables. The results revealed that the main effects were significant in both dimensions (*F*s(2, 874) > 867.997, *p*s < 0.001, partial *η*^2^s > 0.664), and Tukey's posthoc comparisons showed significant differences among all the clusters (*p*s < 0.01).

The first cluster (517 respondents) was distinctive because the first dimension was average, whereas the second dimension had negative values. This group was named the *preventative group* because it was inferred that they mainly took measures to prevent or remove objects that could have led to unintentional injuries in their children. The second cluster (173 respondents), with a negative value for the first dimension and a positive value for the second dimension, it was considered a group that mainly takes measures to explicitly prevent children from approaching or contacting objects and then maintains this state. Accordingly, this group was named the *constantly restrictive group*. The third cluster (185 respondents) had positive values for both the first and second dimensions, especially the first dimension. This was a distinctive feature that differentiated it from the other two groups. Thus, it suggested that this group mainly comprised parents who took measures to prevent their children from approaching or contacting the dimensions on a case-by-case basis. Therefore, this group was referred to as the *case-by-case restrictive group*.

### Association with family socioeconomic background

A preliminary analysis was conducted on the association between cluster classification and family socioeconomic background. Further, there were no differences in family structure, parental education, employment, or income.

### Association with children’s demographic variables

It was examined whether the distribution of clusters differed according to the age of children (Table 3). The age bias was significant (*χ*^2^ = 106.890, *df* = 12, *p* = 0.000, Cramer's *V* = 0.247), and residual analysis showed that the first cluster was more common in 6–11-month-olds, the second cluster in the 1–2 year age range, and the third cluster in the ≥ 4 years age range.

**Table 3:** Distribution of the clusters by age groups

	Cluster		
Age	1	2	3
6―11 mo.	46	18	1
12―23 mo.	64	39	6
24―35 mo.	80	41	11
36―47 mo.	86	26	31
48―59 mo.	85	15	38
60―71 mo.	75	24	46
72 mo.―	81	10	52
*N* = 875			

An association with birth order (first, second, or later) was also found (Table 4, *χ*^2^ = 8.046, *df* = 2, *p* = 0.018, Cramer's *V* = 0.096). The second cluster was more common in first-born children, and the third cluster was more common in second- and later-born children. No association was found between the cluster classification and the child's sex (*χ*^2^ = 0.560, *df* = 2, *p* = 0.756, Cramer's *V* = 0.025).

**Table 4:** Distribution of the clusters by birth order

	Cluster		
Birth order	1	2	3
First-born	201	100	96
Second-born or subsequent	248	84	146

*N* = 875

### Constructs of child- and mother-related variables

Since the confirmatory factor analysis results of children's temperament did not meet the general criteria, an exploratory factor analysis was conducted (see Supplementary File 2). Careful consideration revealed that the temperament scale used in this study consisted of two factors: *inhibitory tendency* and *negative emotion*. The mean scores of these two factors were used in the subsequent analyses.

In contrast, the original study on life habits and behaviors did not specifically identify any background factors, and an exploratory factor analysis was conducted. The results of the factor analysis using promax rotation with the maximum likelihood method showed that factors with eigenvalues of one or more were the focus. Given that the value of the first factor was exceptionally high (7.204, 1.456, and 1.142, respectively), it was considered appropriate to assume a one-factor structure. The reliability coefficients for all 18 items were as high as 0.899, and the mean scores for these scales were used in the analysis.

For mothers'child-rearing attitudes, a confirmatory factor analysis was conducted based on the original study but failed to meet the criteria. Therefore, an exploratory factor analysis was conducted (Supplementary File 3). Consequently, treating them as a single factor was appropriate, with several items that were scored and used in subsequent analyses.

### Measures against unintentional injuries and their associations with other variables

It is necessary to understand which variables have the most significant influence on the measures parents take to protect their children from unintentional injuries. Thus, a multinomial logistic regression analysis was conducted using all five variables as explanatory variables. The variables included age, birth order, temperament, independence in life habits and behaviors, and mothers'child-rearing attitudes. The results showed that the model's goodness of fit was significant (*χ*^2^ = 143.692, *df* = 22, *p* = 0.000). As shown in Table 5, families with younger children were more likely to be classified in Cluster 2 and, conversely, less likely to be classified in Cluster 3. The second cluster was related to independence in life habits and behaviors, and the third cluster was related to mothers'child-rearing attitudes.

**Table 5:** Multinomial logistic regression analysis on the association between home-safety practices and other variables (cluster 1 as reference category)

Categories	Odds ratio	95%CI lower	95%CI upper	*p*
Cluster 2				
Age grade ^a^: 6–11 mo.	7.005	2.398	20.464	<.001
12–23 mo.	9.661	3.845	24.276	<.001
24–35 mo.	5.796	2.573	13.06	<.001
36–47 mo.	2.963	1.317	6.662	0.009
48–59 mo.	1.591	0.668	3.788	0.295
60–71 mo.	2.613	1.167	5.848	0.019
72 mo.–	1.000			
Birth order ^b^: first-born	1.195	0.833	1.713	0.334
second-born or subsequent	1.000			
Temperament: inhibitory tendency	1.118	0.876	1.426	0.370
negative emotion	0.927	0.693	1.240	0.610
Self-reliance	2.046	1.192	3.510	0.009
Parental attitude that encourages a child's challenge	1.067	0.723	1.575	0.744
Cluster 3				
Age grade ^a^: 6–11 mo.	0.032	0.004	0.259	0.001
12–23 mo.	0.140	0.05	0.392	<.001
24–35 mo.	0.214	0.098	0.469	<.001
36–47 mo.	0.611	0.347	1.075	0.088
48–59 mo.	0.788	0.461	1.344	0.382
60–71 mo.	0.988	0.592	1.651	0.965
72 mo.–	1.000			
Birth order ^b^: first-born	0.813	0.566	1.167	0.262
second-born or subsequent	1.000			
Temperament: inhibitory tendency	1.046	0.810	1.351	0.732
negative emotion	0.932	0.702	1.237	0.626
Self-reliance	0.622	0.362	1.069	0.086
Parental attitude that encourages a child's challenge	1.838	1.241	2.721	0.002
*χ*^2^ value	143.692			
− 2 log-likelihood	1534.766			
Cox-Snell *R*^2^	.151			

*N* = 875

^a^72 mo.– as a reference category

^b^second-born or subsequent as a reference category

## Discussion

This study aimed to clarify how parents of infants and young children implement measures to avoid unintentional injuries at home, their typology and changes with increasing age, and to determine the effects of demographic variables such as sex, birth order, temperament characteristics of the children, self-reliance in life habits and behaviors, and mothers'child-rearing attitudes. Unintentional injuries are among the leading causes of death among children in many countries and are of great concern worldwide [1]. Most of these injuries are thought to be avoidable through parental behavior [19], and their effectiveness has been confirmed [20]. However, there is limited data from Asian countries, especially Japan, and there have been few studies on the classification of measures against unintentional injuries and their relationships with various variables. It is natural to expect that the types of measures change as children grow. Morrongiello et al. [56] noted parents gradually relax their monitoring and prohibitions; however, little research has accurately captured the process of this change. Furthermore, it has not been clarified how children's behavioral characteristics and mothers'child-rearing attitudes are related to the measures taken against unintentional injuries and how they change with age.

The correspondence and subsequent cluster analyses showed that the countermeasures against unintentional injuries could be classified into three major groups. The first cluster was characterized by parental efforts to keep objects that could lead to unintentional injuries out of the reach of children (i.e., to place only safe objects around children), which was the most common (59.1%). The second cluster was characterized by explicit parental efforts to block the approach and contact of children (i.e., to block it with something so that they could not engage in it), and this condition was maintained regularly rather than temporarily. Furthermore, parents in the third cluster did not completely block their children from touching dangerous objects or approaching hazardous places. Nonetheless, they instead took precautions to avoid unintentional injuries on a case-by-case basis (e.g., covering the toilet lids after use and draining the bathtub immediately after bathing).

Classification into the above three clusters was primarily related to the children's age, with those under one year of age in the first cluster, those aged 1–2 years in the second cluster, and those four or older in the third cluster. Over half of the children in all age groups were in the first cluster. Further, it was confirmed that measures to prevent children from engaging in hazardous places or objects shifted from explicit and routine measures to those taken by parents on a case-by-case basis as children's age increased. From the parents'perspective, explicitly and constantly blocking or closing a particular place or object is the most reliable countermeasure. Simultaneously, this approach is detrimental to the parents'smooth daily activities and convenience since items such as child guards, locks, and outlet covers are taken at the expense of parents'daily activities and impair their freedom of movement. However, this countermeasure may have an educational impact on children; they can visually confirm that the place is dangerous (object). Therefore, the parents in the third cluster may have intended to provide their children with learning opportunities. In particular, this was to avoid risks by partially exposing them to objects blocked from contact or access while managing extremely hazardous objects and places on a case-by-case basis.

An analysis of the relationship with demographic variables showed that the measures differed according to birth order, with the second cluster being more common for first-born children and the third cluster being more common for second-born or later children. Generally, parents tend to be more careful and attentive to their involvement with their first child and exercise less restraint on their children's behavior with their second or subsequent children [57]. Japanese data also show that parents of first-born children tend to be concerned about many things, including physical development and illness [58]. Therefore, parents should be cautious and take safe measures for their first child. A possible reason why the third cluster included more second or subsequent children may be that the experience of raising older children may have made the parents feel more comfortable and less restrictive with their children. Another possibility is that parents may have taken measures against accidents at home based on the developmental status of their older children. Future studies should address these limitations.

Multinomial logistic regression analysis was conducted with the cluster as the target and demographic variables, child-related variables (temperament, life habits, and behavioral independence), and mother-related variables (child-rearing attitude) as explanatory variables. The results revealed that families with younger children were more likely to be classified in Cluster 2 and, conversely, less likely in Cluster 3, consistent with previous descriptions. Furthermore, the independence of children's life habits and behaviors affected their classification into a second cluster. Notably, parents explicitly closed hazardous places or objects to deter their children from approaching and contacting them where the risk of injury is expected. This finding contradicts intuitive expectations because parents of children with relatively independent life habits and behaviors may tend not to keep an eye on their children, as parents can leave such children without concern. Given this, parents of children with independent life habits may close or block hazardous places and objects, intending to spend more time away from their children and feel more at ease. Moreover, explicitly showing children more self-reliance on dangerous parts of the house could help them understand where to avoid risks.

No association was found between the children’s temperament and measures taken to prevent accidents. Previous studies have confirmed the influence of children’s temperament on the occurrence of unintentional injuries, where children with characteristics, such as high activity and stimulus-seeking behavior were more likely to experience unintentional injuries [29–31, 59]. The data of this study differ from those of previous studies in that it was not about the actual occurrence of accidents, but about measures to prevent accidents. Although there are limited findings on the association between perceived physical ability and temperamental characteristics of children and strategies parents take to prevent unintentional injuries [60], it can be said that the present study extends previous findings by examining several types of strategies for injury prevention and their associations with child temperament. Nevertheless, only two temperaments of children were considered in this study: inhibitory tendencies and negative emotions. Therefore, it is necessary to use a more comprehensive temperament scale to confirm the reproducibility of the results of the present study.

Regarding mother-related variables, mothers who actively encourage their children's independence are more likely to avoid danger on a case-by-case basis. This is as opposed to explicitly and routinely discouraging their children from approaching places or touching objects that might cause injury. Letting children do something while knowing there is a risk increases the likelihood of injury. Nonetheless, parents may try to nurture their children's ability to protect themselves by implementing necessary countermeasures on a case-by-case basis while monitoring their children's behavior.

## Conclusions

The results of this study confirmed the general process by which countermeasures against children's unintentional injuries at home proceed from (1) the phase in which parents protect their children by keeping potentially hazardous objects away from them. (2) The phase in which parents prevent unintentional injuries by blocking contact and accessing hazardous objects and places. (3) The phase in which parents open some hazardous environments to their children but simultaneously monitor their children's behavior and respond to prevent injuries on a case-by-case basis. The data also showed that child-related variables (self-reliance on children's life habits and behavior) and parent-related variables (mothers'child-rearing attitudes) were associated with the countermeasures taken by the parents. According to the existing conceptual framework, it has been pointed out that three major factors are involved in the occurrence of children's unintentional injuries: child factors, environmental factors, and parental factors. However, very few studies have addressed the classification of preventive countermeasures and their change over a more extended age range, contributing to the extension of research in this field.

However, the cross-sectional design of this study did not provide substantial evidence on the mechanisms underlying the transition from one phase to the next or on the factors that produce individual differences. Longitudinal data should be used to elucidate the detailed mechanisms in the future. In addition, more comprehensive measures concerning children's temperaments and parents'child-rearing attitudes should be considered in future studies.

## Supplementary Information

Supplementary Material 1.

## Data Availability

Data cannot be shared publicly because of ethics committee restrictions; however, materials and analysis codes are available upon request by contacting the corresponding author via e-mail.
