# Supplementary material for: Classification of unintentional injury prevention practices for infants and young children at home: developmental process and associations with other variables in Japanese families
Source: BMC Psychol. 2025 Apr 25;13:441. doi: 10.1186/s40359-025-02770-5 (PMC12023365; doi:10.1186/s40359-025-02770-5)
Supplement: Supplementary file 1 — Supplementary Material 1. [file 40359_2025_2770_MOESM1_ESM.docx]

**Supplementary files**

**Supplementary file 1:** *Validity of the scale on an injury prevention strategy*

The “Accident Prevention Handbook” published by the Child and Family Agency classifies unintentional accidents parents of preschool children (0–6 years old) should pay attention to six categories and present 5–11 subcategories for each. The table below shows the number of subcategories for each of the five categories, except for “car-related accidents.” This is not the main topic of this study, nor is the age at which parents should be aware of those accidents. When divided into two groups, those to which parents should pay attention from 0 years of age and those to which parents should do so from 1 year of age onward, 25 (62.5%) of the 40 subcategories were classified into the former group. Among the 15 items parents should be aware of after the age of 1 year, eight were outdoor accidents. A breakdown of the 15 items that parents should be mindful of after the age of 1 year revealed that seven of these were indoor accidents related to the topics of this study. These seven accidents were: a. Fall from balconies (1 year old or older), b. Fall from windows or bay windows (1 year old or older), c. Hands or fingers caught in doors or windows (1–3 years old), d. Injuries with a knife in the kitchen (1–6 years old), e. Getting trapped under fallen furniture such as a chest of drawers (1–6 years old), f. Accidents involving front-loading washing machines (1–6 years old), g. Throat in injuries caused by toothbrushes or other injuries when brushing the teeth (1–6 years old). Careful examination of the items on the injury prevention scale in this study revealed that this scale covers measures for five accidents (Category 21 for a and b, Category 13 and 14 for c, Category 24 for d, and Category 8 for e; see Table 2), except for f and g. Furthermore, when the 31 items of this scale were re-examined based on the “Accident Prevention Handbook,” it was confirmed that the scale adequately covers accidents at home. Therefore, although the injury prevention scale items developed in this study were derived from a study of families with children < 1.5 years old, the scale should be expanded to include families with children aged < 7 years.

**Accidents that occur in preschool children**

|  |  |  | Age at which caution begins to be needed | |
| --- | --- | --- | --- | --- |
| Accidents | No. of subcategory |  | 0 yrs. old | 1 yrs. old ^a^ |
| Choking and accidental ingestion | 8 |  | 8 | 0/0 |
| Falls | 11 |  | 6 | 3/2 |
| Water-related accidents | 6 |  | 3 | 3/0 |
| Burn-related accidents | 5 |  | 4 | 1/0 |
| Caught in things, cuts, and other accidents | 10 |  | 4 | 1/5 |
| Total | 40 |  | 25 | 8/7 |
| ^a^ outdoor or indoor |  |  |  |  |

**Supplementary file 2:** *Constructs of children’s temperament*

Confirmatory factor analysis was conducted on the children's temperament using the maximum likelihood method to assess the factorial validity of the model. However, the results failed to meet the general criteria (*GFI* = .916, *AGFI* = .864, *CFI* =.774, and *RMSEA* =.099). Therefore, exploratory factor analysis was conducted using promax rotation with the maximum likelihood method and by checking the attenuation of the scree plot. Consequently, it was considered appropriate to assume two factors. Nonetheless, some items (items 2, 5, 6, 9, 10, and 12) had low commonality and were eliminated sequentially, yielding the results below. Factor 1 consisted of items 7 (reversal), 8 (reversal), and 4 and was designated an inhibitory tendency. The second factor consisted of items 1, 11, and 3 and was designated a negative emotion. The reliability coefficients of the two factors are .695 and .673, and there was no correlation between them.

**Factor loadings for children's temperament**

|  | Factor loading | |
| --- | --- | --- |
| Factors/Items | 1 | 2 |
| **Inhibitory tendency** |  |  |
| 7. Be in a good mood when played with by strangers ^r^ | **.901** | -.012 |
| 8. Speak to strangers immediately ^r^ | **.649** | -.141 |
| 4. Cry when someone they have never met before touches their body | **.484** | .290 |
| **Negative emotion** |  |  |
| 1. Short-tempered and angry | -.048 | **.737** |
| 11. Express intense emotions when things don't go their way | -.055 | **.686** |
| 3. Have moody days that linger all day | .078 | **.515** |

^r^ reversal items

**Supplementary file 3:** *Constructs of mother’s child-rearing attitude*

Confirmatory factor analysis based on the factor structure reported in an original article resulted in *GFI* = .927, *AGFI* = .861, *CFI* = .656, and *RMSEA* = .120, which were below the criteria. Therefore, exploratory factor analysis was conducted using promax rotation of the maximum likelihood method, with the number of factors fixed at 2, as the original scale. However, five of the eight items showed relatively high loadings on the first factor (.664, .569, .454, .349, .344) and two items on the second factor ( .654, .491), as shown in Table below. Therefore, the five items loaded highly on the first factor were scored and used in the analysis. This strongly indicated encouraging children to challenge themselves, even if it was somewhat dangerous. The alpha coefficient was .613.

**Factor loadings for children's temperament**

|  | Factor loading | |
| --- | --- | --- |
| Factors/Items | 1 | 2 |
| **Factor 1** |  |  |
| 8. Let the child do what they want to do even if it is a little unskilled or dangerous | **.664** | .193 |
| 5. Let the child help out at home | **.569** | .051 |
| 6. tolerate minor injuries and dangers to the child | **.454** | .055 |
| 4. Let the child prepare things to take to school or preschool by themselves without parents checking or helping | **.349** | -.321 |
| 7. Let the child go alone when going to the bathroom at night | **.344** | -.281 |
| **Factor 2** |  |  |
| 3. At mealtime, cut up fish and meat into bite-sized pieces so that the child can eat easily | .009 | **.654** |
| 2. Help the child when things slow down or don't seem to go well, if allowed to do it alone | .116 | **.491** |
| 1. See a doctor even in case of minor illness or injury | .074 | .225 |
